# How to Establish a Minimal Invasive and Stable Carotid Artery Stenosis Rabbit Model? A Simple and Effective Carotid Artery Balloon Strain Technique

**DOI:** 10.3389/fphys.2021.752716

**Published:** 2021-11-04

**Authors:** Zhengli Liu, Maofeng Gong, Boxiang Zhao, Jianping Gu, Haobo Su, Yangyi Zhou, Guanqi Fu, Xu He, Jie Kong

**Affiliations:** Department of Interventional Radiology, Nanjing First Hospital, Nanjing Medical University, Nanjing, China

**Keywords:** common carotid artery stenosis, injury, fluoroscopy guidance, balloon strain, rabbit model

## Abstract

**Background:** The objective of this study is to establish a minimally invasive technique to create a stable carotid artery stenosis rabbit model. This article summarizes the specific methods and key points of this technology.

**Methods:** The experiment studied a rabbit that was anesthetized through the vein. After the femoral artery was exposed, a minimally invasive needle was used to puncture the femoral artery, then the sheath was placed into the artery. We primarily put a catheter in the ascending aorta for angiography and then used a PT2 guidewire for super-selection. The PT2 guidewire was retained, and a balloon was placed in the right common carotid artery (CCA) through a guidewire to inflate it three times. Six rabbits in the 2- (2W) and 4-week (4W) groups were examined at 14 and 28 days, respectively. The rabbits in the control group received angiography at the beginning and 28 days later but without balloon injury. After angiography assessment, specimens of right CCA were dissected. Pathological and immunohistochemical examinations were performed on the collected specimens, and iFlow analysis was performed as well.

**Results:** All the 18 animals which survived were observed. The rabbits in the 2W and 4W groups showed stenosis of the right CCA. Digital subtraction angiography showed the diameter was lower than that in the control group (1.04 ± 0.1, 0.71 ± 0.12, and 1.83 ± 0.08 mm in 2W, 4W, and control group, *P* < 0.05). Pathology also suggested carotid stenosis and obvious intimal hyperplasia. The results of immunohistochemistry showed that α-smooth muscle actin was highly expressed in the 2W and 4W groups, and the integrated optical density (IOD) value was higher than that in the control group (14,807.11 ± 1,822.3, 22,245.96 ± 1,212.82, and 6,537.16 ± 1,186.62 in the 2W, 4W, and control group, *P* < 0.05). Meanwhile, a cluster of differentiation 31 (CD31) was low expressed in the 2W and 4W groups, and the IOD value was lower than that in the control group (519.14 ± 44.4, 1,029.64 ± 98.48, and 1,502.05 ± 88.79 in the 2W, 4W, and control group, *P* < 0.05), which suggested endothelial damage and partial repair. The analysis by iFlow showed that the time-to-peak after balloon strain in the 2W and 4W groups were longer than that in the control group.

**Conclusion:** We established a minimally invasive, effective, and safe method to establish a carotid artery stenosis rabbit model. The highlights of this technology were the application of minimally invasive methods, reducing surgical bleeding, infection, and related complications. This technology avoided the influence of tissue around CCA in the traditional carotid artery balloon injury model, which might lead to more accurate treatment outcomes.

## Introduction

According to statistics from the American Heart Association in 2020, more than 795,000 people experience a first or recurring stroke each year. Among these people, 87% showed ischemic stroke, 10% showed cerebral hemorrhage, and another 3% showed subarachnoid hemorrhage of all the strokes (Go et al., 2014). From 1995 to 2012, the hospitalization rate of 18- to 44-year-old male patients with ischemic stroke almost doubled (George et al., 2017). Among the patients, 25–30% of ischemic strokes are closely related to carotid arteriosclerosis (). Whether there is a transient ischemic attack (TIA) or other neurological symptoms in the past 6 months, common carotid artery stenosis can be divided into symptomatic common carotid artery stenosis and asymptomatic common carotid artery stenosis. Related risk factors for common carotid artery stenosis include high blood pressure, hyperlipidemia, glucose metabolism disorders, obesity, lack of exercise, sleep apnea, and many other reasons (Furie et al., 2011). The surgical treatment of common carotid artery stenosis mainly includes carotid endarterectomy (CEA) and carotid artery stent (CAS) (Cronenwett and Johnston, 2014). Non-surgical treatments include antihypertensive drug therapy (Brott et al., 2016), diabetes drug therapy (Brott et al., 2016), lipid-lowering drug therapy (Brott et al., 2016), antiplatelet and anticoagulant therapy (BonatiL et al., 2015), improving metabolic status (Furie et al., 2011), and other treatment methods. In order to accelerate the hemodynamic analysis of the common carotid artery stenosis and the evaluation of various treatment strategies, it is necessary to establish a reproducible rabbit common carotid artery stenosis model. Previous studies have established some methods for rabbit common carotid artery stenosis models, including surgical sutures of rabbit common carotid artery (Golino et al., 1992). Other methods have also been reported such as inserting a guidewire from the external carotid artery of the rat and then retrogradely entering the common carotid artery for balloon strain (Tsuruta et al., 2007). However, traditional surgical methods are complicated and traumatic, and it is difficult to assess the degree of stenosis of the common carotid artery in a quantitative way. The rabbit model of high-fat diet-induced atherosclerosis requires long-term high-fat feeding of animals, and the uncertainty of the model is high (Zha et al., 2019). In this study, we reported a minimally invasive and stable method, including technical characteristics, hemodynamic changes, and pathological and immunohistochemical characteristics of the stenosis of the stable carotid artery stenosis rabbit model.

## Materials and Methods

### Animal

All experimental procedures were approved by the Animal Care and Use Subcommittee at Nanjing Medical University (IACUC OF NMU). The experiment used 18 New Zealand White rabbits (Yizheng Anlimao Biotechnology Co. Ltd., Yangzhou, China, License number SCXK2016-0005) weighing between 1.5 and 2 kg. After the experiment, the animals were raised in the Animal Center of Nanjing First Hospital, Nanjing Medical University.

### Method of Operation

Rabbits in this research were anesthetized by injecting pentobarbital sodium injection (50 mg/ml, 0.4–0.6 ml/kg, New Asia Pharmaceutical, Shanghai, China) through the rabbit marginal auricular vein slowly. The rabbit was in the supine position, and the skin was prepared locally in the right groin area and sterilized with 70% medical alcohol. The rabbit fur on the right groin area was removed to prepare for puncture.

Firstly, we took 5% lidocaine (20 mg/ml, Hebei Tiancheng Pharmaceutical Co, Ltd., Cangzhou, China) to locally anesthetize the right inguinal area of the rabbit. We took an incision of about 2 cm in length and separated layer by layer until the rabbit femoral artery was exposed. Two silk sutures were placed below the femoral artery at the proximal and distal ends, respectively. We used a 21G minimally invasive puncture needle (MeritMedical, Rockland, MA, United States) to puncture the exposed femoral artery. The micro-guide wire was introduced from the tail of the needle when the blood gushed from the tail of the needle.

Afterward, we confirmed that the micro-guide wire has entered the abdominal aorta by fluoroscopy, and then the 4 F Prelude Sheath Introducer (MeritMedical, Rockland, MA, United States) was placed into the femoral artery through a micro-guide wire. The PT^2^ guidewire (0.03556cm/260cm, Boston Scientific, Marlborough, MA, United States) combined with 2.4F Microcatheter (MeritMedical, Rockland, MA, United States) and VER 135°Angiographic Catheter (Cordis, Miami Lakes, FL, United States) were used to reach the ascending aorta to do the aortic arch angiography.

The contrast medium was iodixanol (Visipaque, 320 mg/ml, GE Healthcare, Ireland), and the injection rate was 2 ml/s with a total amount of 6 ml. The radiography could clearly show the brachiocephalic trunk and the left subclavian artery. The left and right common carotid arteries were separated from the brachiocephalic trunk. Then the VER 135°Angiographic Catheter was slightly retracted to put its head in the brachiocephalic trunk. The right common carotid artery was super-selected with PT^2^ guidewire and microcatheter. The angiography used to confirm carotid artery was at the rate of 0.5 ml/s, and the total amount of 2 ml. The micro-guidewire was kept, the VER 135° Angiographic Catheter and Microcatheter were withdrawn, and the rapid exchange balloon dilatation catheter (3.5 mm × 12 mm, Boston Scientific, Marlborough, MA, United States) was replaced. The mark before the balloon was located at the mandibular angle. A pressure pump (Medtronic, Minneapolis, Minnesota) was used to pressurize the balloon until inflating it completely. Then, the pressure of the balloon was maintained at 6 atm, slowly withdrawing the balloon downward so that the mark was located at the clavicle head, and then the balloon was deflated and its tip was sent to the mandibular angle three times. Then, the balloon was withdrawn and replaced with the VER 135°Angiographic Catheter and Microcatheter for the angiography of the right common carotid artery and aortic arch at the same rate and amount (2 ml/s, 6 ml). Six rabbits were randomly selected in the 2W group and six rabbits in the 4W group received balloon strain, while six rabbits in the control group only received angiography. Six rabbits in the 2W group and six rabbits in the 4W group received balloon strain, while six rabbits in the control group only received angiography. Finally, the catheter was withdrawn, the femoral artery was ligated to prevent bleeding, and a WEGO Silk suture (non-absorbable suture, Shandong Weigao MedicalGroup Co, Ltd., Weihai, China) was used to suture the groin area of the rabbits. After the operation, each rabbit was injected intramuscularly with 200,000 IU of penicillin (penicillin sodium; Shandong Lukang Pharmaceutical Co, Ltd., Jining, China) to prevent infection.

### Angiographic Review Evaluation

The angiographic review evaluation was carried out on the 14th and 28th days after the operation. The carotid artery angiography reexamination was used to evaluate whether there is vascular stenosis. The contrast rate was 2 ml/s and the total amount was 6 ml. The 12 rabbits in the experimental group were divided into 2 (2W) and 4-week (4W) groups. The six rabbits of the 2W group received angiography 14 days after the operation, and the six rabbits in the 4W group received the same evaluation after 28 days. The six rabbits in the control group received angiography only and did not undergo balloon injury. Finally, we evaluated the angiography images of the three groups of rabbits.

### Anatomy and Histology

After the angiography review was completed, the rabbits were sacrificed and dissected, and specimens of right common carotid arteries were collected. The pathology sections were required from the middle to the narrow section, about the midpoint between the clavicle head and the mandibular angle. The cross-sectional sections of CCA were examined by H&E staining. We used software CaseViewer 2.4 (3DHISTECH Ltd., Budapest, Hungary) to measure the diameter of the common carotid artery. At the same time, the CCA specimens of each experimental group and control group were subjected to immunohistochemical analysis. We observed and analyzed the expression and distribution of α-smooth muscle actin (α-SMA) and a cluster of differentiation 31 (CD31) in the common carotid artery to evaluate the proliferation of smooth muscle cells and endothelial cells. The pictures of CCA immunohistochemistry were analyzed by computer image quantitative analysis software Image-Pro Plus 6 (Media Cybernetics Inc., Rockville, MD, United States). The integrated optical density (IOD) was measured and analyzed, which clarified the pathological process and pathological mechanism.

### Image Post-processing

After the operation, the SIEMENS Syngo workstation (SIEMENS Healthineers, Erlangen, Germany) was used for image post-processing. The angiographic images of the aortic arch were analyzed by Syngo iFlow VC21 of the SIEMENS Artis Zee post-processing workstation. After the ruler was calibrated, the diameters of the right common carotid arteries were measured. The stenosis rate was calculated as the diameter difference of the right CCA before and after balloon injury divided by the diameter of the right CCA before balloon injury. Syngo iFlow VC21 calculated the time interval from the image acquisition to the peak gray value of each point based on the value of each pixel in the digital subtraction angiography (DSA) angiography image, defining the time-to-peak (TTP) to evaluate the speed of local blood flow. Then, the TTP values were measured at the beginning of the right common carotid artery, the proximal 1/3, the distal 1/3, and the bifurcation of the common carotid artery.

### Statistical Evaluation

Statistical analysis was performed with SPSS 22 software (SPSS, Inc., Chicago, IL, United States). The analysis of variance and *t*-test was used to analyze continuous data. *P* < 0.05 is considered of statistical difference.

## Results

### Surgery and Digital Subtraction Angiography Imaging

**FIGURE 1:**
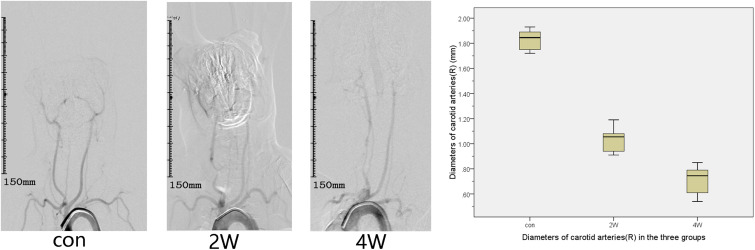
This figure describes aortic arch angiography in the control, 2-week (2W), and 4-week (4W) groups, which suggests the diameter of the right common carotid artery of the rabbit (experimental group) in the 2W group and the 4W group are both indicated significant stenosis compared with the control group.

**FIGURE 2:**
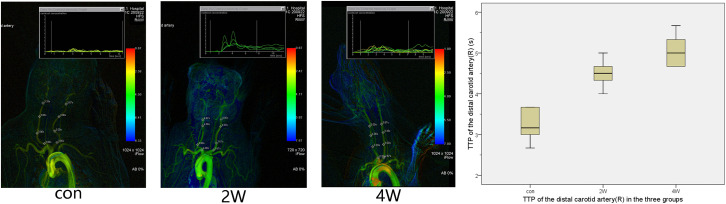
This figure shows the time-to-peak (TTP) in three groups. It is possible to calculate and display flow curves for single pixel markers. After color-coding of TTP, arteries with a shorter time tend to have a warmer color, while arteries with a long time tend to have a colder color. The TTP after balloon strain in the 2W and 4W groups was longer than that in the control group.

The puncture and angiography of all 18 animals were successful, and all animals in the three groups survived to the end of observation during the period. The common carotid artery balloon strain in the 2W group and 4W group were successfully completed. The SIEMENS Syngo workstation was used to measure the diameter of the right common carotid artery three times, and the average value was taken. The diameters of the right common carotid artery in the 2W, 4W, and control groups before the operation were 1.87 ± 0.15, 1.84 ± 0.14, and 1.86 ± 0.07 mm, respectively. At the end of the observation, the diameter of the right CCA in the 2W, 4W group, and control group were 1.04 ± 0.1, 0.71 ± 0.12, and 1.83 ± 0.08 mm, respectively. The length of the stenosis segment in the 2W and 4W groups was 3.38 ± 0.49 and 4.4 ± 0.57 cm, respectively. The exact diameter of the common carotid artery of the rabbit before balloon strain and at the end of observation is shown in Table 1. The diameter of the right common carotid artery of the rabbit (experimental group) in the 2W and the 4W groups were both indicated significant stenosis compared with the control group. Analysis of variance indicated that balloon injury had an effect on the diameter of the rabbit’s right common carotid artery (*P* < 0.05). The preoperative and at the end of observation angiography are shown in Figure 1. The analysis by IFlow indicated that the TTP of the distal end of the right common carotid artery in the 2W group, 4W group, and control groups were 4.5 ± 0.35, 5.06 ± 0.39, and 3.33 ± 0.34 s, respectively, which can be considered as the common carotid artery blood speed in the three groups. The overall mean of TTP was significantly different at *P* < 0.05, which is described in Figure 2.

**TABLE 1:** Digital subtraction angiography (DSA) angiography and gross anatomy results*.

Group	No.	Previous carotid diameter(R)/mm*	End point carotid diameter(R)/mm*
2W	2W1	1.86	1.05
	2W2	1.62	0.94
	2W3	1.80	1.08
	2W4	2.06	1.19
	2W5	1.91	0.91
	2W6	1.99	1.06
4W	4W1	1.81	0.72
	4W2	1.78	0.77
	4W3	1.67	0.61
	4W4	1.79	0.54
	4W5	1.92	0.79
	4W6	2.08	0.85
con	con1	1.86	1.82
	con2	1.90	1.93
	con3	1.79	1.75
	con4	1.82	1.72
	con5	1.80	1.87
	con6	1.97	1.89

*The diameter of DSA is determined by the SIEMENS Syngo workstation, and the diameter of gross anatomy is determined by CaseViewer.

### Gross Anatomy and Pathology

**FIGURE 3:**
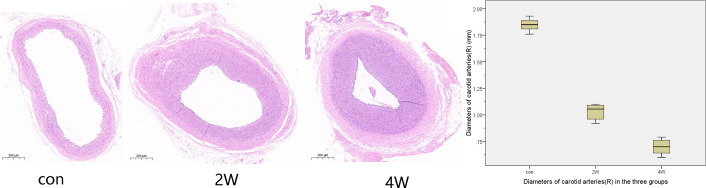
The histological specimens were formalin-fixed and then paraffin-embedded. After paraffin section dewaxing to water, hematoxylin and eosin staining, and dehydration seal, we got the HE staining pathological specimen. This figure shows the cross-sectional pathology of the right common carotid artery in three groups, suggesting that the vascular endothelium of the 2W group and the 4W group has an obvious proliferation of smooth muscle and luminal stenosis. The 4W group has a more prominent proliferation of smooth muscle cells, with a narrower lumen, compared to the control group.

After 14 and 28 days of angiographic reexamination, the animals to be observed were sacrificed and dissected meanwhile. Anatomy showed that all the common carotid arteries on the side of the balloon strain had contractures and stenosis, and obvious proliferation of vascular smooth muscle cells occurred in the lumen. The CaseViewer was used to measure the common carotid artery lumen diameter and lumen area three times and the average of the measurements was taken. The results showed that the diameters of the common carotid artery in the 2W, 4W, and control groups were 1.03 ± 0.07, 0.7 ± 0.07, and 1.85 ± 0.06 mm, respectively, with the areas of 0.81 ± 0.11, 0.42 ± 0.07, and 2.65 ± 0.38 mm^2^, respectively. Figure 3 shows the pathological and the diameter comparison of the three groups, suggesting that the vascular intima of the 2W and 4W groups has an obvious proliferation of smooth muscle, and there was obvious luminal stenosis. While the 4W group has a more prominent proliferation of smooth muscle cells, with a narrower lumen, compared with the control group. The specific pathological pictures and diameter measurements are shown in Figure 3.

### Immunohistochemical Imaging

**FIGURE 4:**
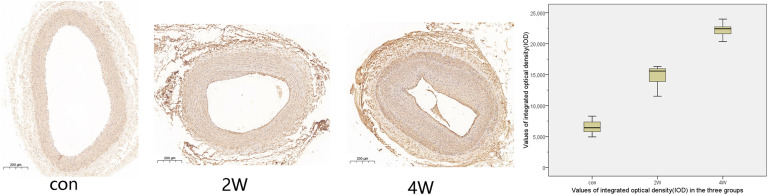
This figure shows the immunohistochemistry and integrated optical density (IOD) value comparison of the right common carotid artery in three groups. α-SMA was low expressed in the control group, but the high expression in the 2W and 4W groups to varying degrees. The darker the color, the stronger the comprehensive positive intensity suggests. The color of the 4W group and the 2W group is darker than that of the control group, suggesting the high expression of α-SMA.

**FIGURE 5:**
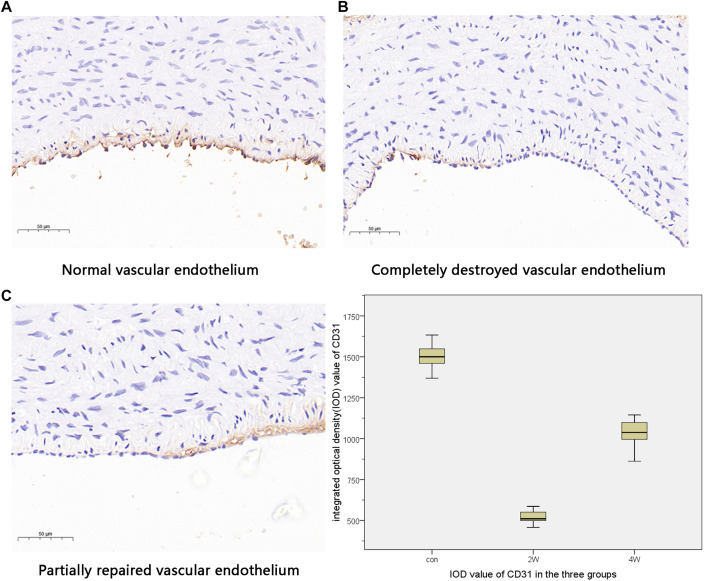
This figure indicates the different states of vascular endothelial injury. The normal vascular endothelium is shown in panels **(A)**. Panels **(B,C)** show different degrees of vascular endothelial damage. The expression of CD31 was the highest in the control group and the lowest in the 2W group, suggesting that the vascular endothelial damage was the most serious in the 2W group and was repaired in the 4W group.

The computer image quantitative analysis technology was used to pre-process the immunohistochemical imaging. α-SMA was low expressed in the control group, but the high expression in the 2W and 4W groups was observed in varying degrees, which was statistically different from the control group at *P* < 0.05). The IOD values measured by Image-Pro Plus were 14,807.11 ± 1,822.3, 22,245.96 ± 1,212.82, and 6,537.16 ± 1,186.62 in the 2W, 4W, and control groups (*P* < 0.05). CD31 was low expressed in the 2W and 4W groups, and the IOD value was lower than that in the control group (519.14 ± 44.4, 1,029.64 ± 98.48, and 1,502.05 ± 88.79 in the 2W, 4W, and control groups, respectively; *P* < 0.05). Additionally, the parameters between the 2W and 4W groups were statistically different at *P* < 0.05. The images of specific immunohistochemistry and the comparison of IOD values of α-SMA and CD31 are shown in Figures 4, 5.

### Comparison of Stenosis Rates

The diameters of the common carotid artery were measured by DSA angiography, and the stenosis rate of the common carotid artery measured by diameter in the 2W group and 4W group was evaluated. The mean stenosis rate of the 4W group was higher than that of the 2W group (44.47 ± 4.46 vs. 61.38 ± 4.69%), and the difference was statistically significant (*P* < 0.05). The stenosis rate of the 2W group and 4W group was within −10 to +10%. The stenosis rate measured by DSA angiography is shown in Table 2.

**TABLE 2:** Common carotid artery obtained by DSA measurement (*n* = 12,%, X ± s).

**Group**	**Stenosis rate (DSA)**
2W (14 days)	44.47 ± 4.46
4W (28 days)	61.38 ± 4.69
*P* value	<0.05

## Discussion

The hemodynamic changes caused by common carotid artery stenosis play an important role in the occurrence of ischemic encephalopathy (Momjian-Mayor and Baron, 2005). The establishment of a suitable animal model of carotid artery stenosis is essential for the research of the occurrence and development of common carotid artery stenosis and the subsequent hemodynamic changes caused by it, which is beneficial for studying the development and mechanism changes of human common carotid atherosclerosis. Since the anatomy of the circulatory system of rabbits is similar to that of humans, this kind of experiment can better simulate the actual situation of humans (Ishii et al., 2006).

In this study, 12 rabbit common carotid artery balloon stenosis models were successfully made by establishing fluoroscopy-guided common carotid artery balloon injury models. The diameter of the common carotid artery on the side of the balloon strain in the 2W and 4W groups was lower than that of the control group. Experiments showed that the common carotid artery on the injured side of the rabbits on the 14th and 28th days has various degrees of stenosis. The mean stenosis rate of the 4W group was higher than that of the 2W group, and there was a statistical difference between the two groups. The stenosis rate of this model is controllable and stable. The IOD value of α-SMA in the 4W group was higher than that in the 2W group, suggesting the proliferation of vascular smooth muscle. On the other hand, the IOD value of CD31 was higher than that of the 2W group, suggesting partial repair of vascular endothelium after injury. Based on the experience in quality control of common carotid artery balloon strain accumulated in this experiment, we provided the following suggestions: (1) the balloon diameter of rabbit common carotid artery strain should not be too large, and the diameter should not exceed over 4 mm of the common carotid artery shown on the angiogram in case of vascular damage, bleeding or even death; (2) the tip of the VER 135° Angiographic Catheter and the microcatheter could be placed on the brachiocephalic trunk to provide sufficient support for the microcatheter and the microcatheter to facilitate the entry of the micro-guidewire and the microcatheter into the common carotid artery; (3) injecting lidocaine of 1 ml before puncturing can reduce the pain of the animal and dilate the femoral artery, thereby increasing the success rate of puncture; (4) aseptic technique was very important, and the puncture site and instruments should be disinfected.

Balloon dilation technology is currently mainly used for the expansion of stenotic vascular, but the diameter of the balloon should be selected to match the lesion site of the vascular stenosis during dilation. Once the balloon is too large, it may cause damage to the vascular intima, inflammatory cell infiltration, a proliferation of smooth muscle in the vascular intima, and cause lumen stenosis (Qi et al., 2018). It has not been reported that fluoroscopy-guided use of balloon strain to establish a carotid artery stenosis model. At present, the common methods of establishing common carotid artery stenosis models include surgical balloon injury direct current stimulation and silk ligation, among others (Betz and Schlote, 1979; Bederson et al., 1986; Chekanov et al., 2002; Huang et al., 2004). Betz E could implant two cap graphite electrodes in the carotid adventitia and stimulate the carotid artery with a constant current (15 ms/impulses, 0.1 mA, 10 Hz). Stimulation was given once every 8–10 h at 28 days cycle. Chekanov VS ligated the exposed carotid artery with a ligation length of at least 3 cm and introduced the balloon to pull it repeatedly three times. However, these methods had a variety of limitations. For example, the surgical balloon injury method required a surgical incision, along with relatively large trauma, and there were certain requirements for micro-suturing, and repeated exercises were required as well. The direct current stimulation method was complicated to operate and the test controllability was poor, and it cannot effectively control the degree of common carotid artery stenosis. The method of silk ligation might lead to the problem that the elastic coefficient of the stenosis differs greatly from that of the actual vessel wall, which was difficult to accurately evaluate.

Compared with ligation, direct current stimulation, and silk ligation, it was more minimally invasive and safer to establish a carotid artery stenosis rabbit model by balloon strain. The main advantages are as follows. First, it could avoid surgical incision in the carotid artery area, has the advantage of being minimally invasive, avoided damaging other vascular during the surgical incision, and eliminated the influence of confounding factors. Second, it could use iFlow software to intuitively and quantitatively make hemodynamics evaluations, which could indirectly help us understand the degree of vascular stenosis. The function of dot delineation of the ROI area in iFlow could ensure that the size of the ROI area remains consistent. Third, through the angiography examination, the change of common carotid artery stenosis could be dynamically assessed over time, and the assessment could be carried out at multiple flexible time points to facilitate follow-up observation. Fourth, it provided convenience for the endovascular treatment model of carotid artery stenosis. In this test, the degree of stenosis of the common carotid artery was evaluated at 14 and 28 days after the operation, and the stenosis rate was stable and controllable.

In general, we used a method to establish a carotid artery stenosis rabbit model, which can cause limited segmental stenosis of the rabbit common carotid artery. This model could be used to study the hemodynamics of carotid artery stenosis and evaluate various treatment options and prognosis of cerebral ischemia.

## Data Availability Statement

The original contributions presented in the study are included in the article/supplementary material, further inquiries can be directed to the corresponding authors.

## Ethics Statement

The animal study was reviewed and approved by Animal Care and Use Subcommittee at Nanjing Medical University (IACUC OF NMU).

## Author Contributions

ZL and MG performed most of the operations. BZ and JG provided a lot of constructive advice in the operation. HS and YZ helped with the software calculation. GF assisted in drawing and revising the pictures. ZL and MG performed draft of the manuscript. XH and JK designed, guided, funded the study, critically revised the manuscript, and final approval for publication. All authors contributed to the article and approved the submitted version.

## Conflict of Interest

The authors declare that the research was conducted in the absence of any commercial or financial relationships that could be construed as a potential conflict of interest.

## Publisher’s Note

All claims expressed in this article are solely those of the authors and do not necessarily represent those of their affiliated organizations, or those of the publisher, the editors and the reviewers. Any product that may be evaluated in this article, or claim that may be made by its manufacturer, is not guaranteed or endorsed by the publisher.
